# Assembling allopolyploid genomes: no longer formidable

**DOI:** 10.1186/s13059-015-0585-5

**Published:** 2015-01-31

**Authors:** Ray Ming, Ching Man Wai

**Affiliations:** FAFU and UIUC-SIB Joint Center for Genomics and Biotechnology, Fujian Agriculture and Forestry University, Fuzhou, Fujian 350002 China; Department of Plant Biology, University of Illinois at Urbana-Champaign, Urbana, IL 61801 USA

## Abstract

A combined approach of whole genome shotgun sequencing and ultra-high density linkage mapping using skim sequencing of a segregating population is effective for assembling allopolyploid genomes.

See related Research, http://dx.doi.org/10.1186/s13059-015-0582-8

## Obstacles to the assembly of allopolyploid genomes

Polyploidy has contributed substantially to the diversification of seed plants and to the extraordinary success of angiosperms, which dominate the earth [1]. Among seed plant species, 35% are polyploids [2]. The ratio of allopolyploidy (from two species) and autopolyploidy (from the same species) is unknown because chromosome counting is insufficient for distinguishing these polyploid cytotypes. Autopolyploid formation is thought to be more frequent than that of allopolyploids [3], but allopolyploids have more advantages during the establishment phase owing to their potential for heterosis; autopolyploids also suffer from reduced fertility [3]. This is consistent with the observation that there are more allopolyploid crops (including wheat, cotton and tobacco, strawberry, and oilseed rape) than there are autopolyploid crops (such as potato, sugarcane and banana).

Allopolyploids are formed by the hybridization of two closely related species, primarily by fertilization of two unreduced gametes or, to a lesser extent, by genome doubling after fertilization of two reduced gametes [3]. The first challenge for assembling allopolyploid genomes is to distinguish between two closely related subgenomes of a species. A second challenge is that, although rare, translocations, duplication and crossovers between homeologous chromosomes (that is, partially homologous chromosomes) do occur. This embedding of fragments of one subgenome into another could result in misassembly of different homeologous chromosomes into one mosaic artificial chromosome. Third, a common feature of plant genomes is that there is often an over-dominance of retrotransposons, which could copy and paste among homeologous chromosomes and potentially cause misassembly.

In this issue of *Genome Biology*, Chapman and colleagues [4] have presented a novel integrative approach to generate a genome assembly of the hexaploid bread wheat genome. Their method combines whole genome shotgun sequencing with ultra-high density linkage mapping achieved by skim sequencing, and the resulting genome quality exceeds that of chromosome arms-based assembly [4].

## How similar are homeologous chromosomes in allopolyploid genomes?

In light of the challenges described above, the whole genome shotgun approach appears not to be applicable for assembling allopolyploid genomes. Traditionally, the approach for sequencing allopolyploid genomes of crop plants has been to sequence progenitor diploid genomes, as done for cotton, strawberry, coffee and oilseed rape. However, the progenitor genomes of the 17 gigabase (Gb) wheat genome are larger than any of the allopolyploid genomes mentioned above, and sequencing any of the three 5.5 Gb progenitor genomes requires substantial investment [5]. The wheat genome comprises 21 large and distinguishable chromosomes, and the wheat community took an approach of sorting each chromosome or chromosome arm for sequencing and assembly [6]. Using this approach it is possible to eliminate misassembly of homeologous chromosomes. However, chromosome sorting does not yield sufficient quantities of DNA for high-throughput sequencing using Illumina technology. It is therefore necessary to amplify each chromosome or arm in short fragments, making it impossible to construct large-insert jumping libraries for scaffolding, which results in short contigs in the assembled genome [6]. This makes subsequent genomic research less efficient.

These challenges demonstrate the significance of the *Genome Biology* study by Chapman and colleagues, which combined whole genome shotgun sequencing and ultra-high density linkage mapping to assemble an allopolyploid genome. ‘Synthetic W7984’ was generated by crossing a tetraploid wheat AABB genome with the diploid DD genome, followed by chromosome doubling, resulting in a contemporary reconstitution of hexaploid wheat. This homozygous line was sequenced at 30× coverage using a whole genome shotgun approach with 2 × 150 base pair (bp) sequences of Illumina TruSeq libraries in paired-end and mate pairs of 250 bp to 4.5 kb in size [4]. The genome of ‘W7984’ was assembled using an enhanced version of Meraculous, a new algorithm for *de novo* genome assembly with deep paired-end short reads [7]. Analysis of 51-mers revealed that no genomic features were present in double or triple copy, indicating the three sets of homeologous chromosomes were separated in the genome assembly. Simulation of 81-mer sequences yielded substantially higher fractions of unique sequences in the genome than that of 51-mers, implying that increasing the sequencing depth further improved the quality of the assembled genome. Identical exons were assembled into the correct subgenomes using information from more divergent flanking intronic and intergenic sequences, a key feature of allopolyploid genomes.

**Figure 1:**
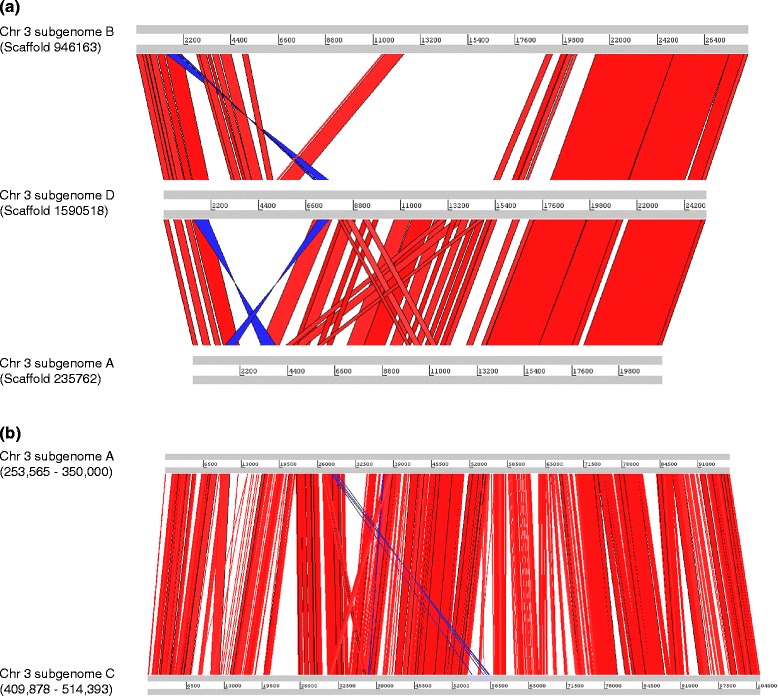
**Collinearity of subgenomes in hexaploid bread wheat and allotetraploid oilseed rape. (a)** Three homeologous regions from subgenomes A, B and D of bread wheat *Triticum aestivum* line Synthetic W7984 were aligned for direct sequence comparison [4]. Scaffold 946163 (position 27,539 to 55,967) of subgenome B, scaffold 1590518 (position 23,362 to 48,554) of subgenome D, and scaffold 235762 (position 75,800 to 97,583) of subgenome A were aligned. Collinear and inverted regions are represented by red and blue lines, respectively. For subgenomes A and D, but not B, a high degree of collinear genome duplication is observed in the central region. **(b)** Two homeologous regions from *Brassica napus* subgenome A (position 253,565 to 350,000) and subgenome C (position 409,878 to 514,393) were aligned for comparison. There were 4,331 SNPs (5.7%) within a 76 kb aligned region. A total of 32,411 bp (14.1%) InDels were detected and the length of the longest one is 4,808 bp. High collinearity between the two subgenomes was observed with minor inversion and duplication events.

The level of DNA sequence identity of homeologous chromosomes in allopolyploid genomes had been a mystery, but draft genomes of hexaploid wheat and tetraploid oilseed rape have now been published [6,8]. Direct comparison of homeologous sequences revealed substantially higher sequence divergence than previously assumed. In bread wheat *Triticum aestivum*, a region compared in chromosomes 3A, 3B and 3D had collinear sequences of 21,784 bp, 28,429 bp and 25,193 bp, respectively. Pairwise comparison between subgenomes A and B, A and D, and B and D revealed 23.3%, 13.5% and 12.8% insertion-deletion (InDel) differences, and 4.5%, 5.2% and 6.1% SNPs in gapless comparison, respectively. The combined DNA sequence differences between subgenomes among these three pairs were 27.8%, 18.7% and 18.9% (Figure 1a). In allotetraploid oilseed rape *Brassica napus*, a 96,436 bp region of subgenome A was collinear to a larger region at subgenome C (104,516 bp), showing an 8.4% InDel difference and 5.7% SNPs in gapless comparison. The combined DNA sequence difference between subgenomes A and C was 14.1% (Figure 1b). Thus, the DNA sequence divergence among homeologous chromosomes ranged from 14.1% to 27.8% in the allopolyploid genomes of *T. aestivum* and *B. napus*, sufficiently different for *de novo* genome assembly of allopolyploid genomes.

## Ultra-high density linkage mapping by skim genome sequencing is indispensable for proper assembly of allopolyploid genomes

Most draft genomes sequenced to date are anchored to a linkage map, typically comprising several hundred or several thousand markers, which is sufficient for anchoring compact diploid genomes that are assembled into large scaffolds. However, for a large hexaploid wheat genome with numerous small scaffolds, conventional high density linkage maps are insufficient for anchoring the majority of scaffolds to chromosomes. Skim sequencing at low coverage of individual genomes from a mapping population is necessary to generate hundreds of thousands of markers for anchoring scaffolds in the correct order and orientation to chromosomes. The best mapping population is a segregating F_1_ population, which can be used to map the meiosis events of the previous generation within each parental genome, without integration of the two parental genomes through recombination in F_2_ populations. Chapman and colleagues generated an ultra-high density map by sequencing 90 individual genomes of a double haploid F1 population at 1.4× coverage, yielding an assembled hexaploid wheat genome that exceeded the quality of an assembly created using a chromosome arm-based approach [4].

The polymorphism rate between the cultivars ‘W7984’ and ‘Opata’ is 0.32%, which would be considered low if this measure was based on DNA markers. However, based on genome sequences, it is more than three markers per kilobase of sequence, outnumbering potential haplotype blocks of any genome by a large margin. Chapman and colleagues tested both reference genome-based and assembly-independent approaches for calling SNPs. An order of magnitude more SNP markers were generated by the reference genome-based approach (19.0 million versus 1.7 million SNPs) and the resulting two genetic maps are identical. Based on their results, reference genome-based SNP calling is better, easier and preferable. With such an ultra-high density genetic map, with 112,687 SNP markers, they were able to identify 10 individuals with a partial or complete loss of a chromosome arm, hence purging them from the mapping population. They also identified regions of segregation distortion in chromosome 6AS and 6DS in ‘W7984’ and chromosome 4DL in ‘Opata’.

A critical application of this ultra-high density linkage map is the validation and correction of assembled scaffolds. For an assembled genome of 9.1 Gb with a genetic map at 2,826 centimorgans, the average number of nucleotides per centimorgan is 3.2 Mb. However, crossovers occur in only 13% of the wheat genome, with a range of 400 kb to 1.2 Mb per centimorgan [4]. In a draft genome with a scaffold N50 at 21.1 kb, SNP markers in a scaffold smaller than 400 kb should be co-segregating with an identical or nearly identical genotype, and an identical genotype for the 87% of the genome with no crossover events. An SNP marker at the end of a scaffold with a different genotype indicates internal errors within the assembled scaffold. Based on this principle, Chapman and colleagues identified and corrected errors at 0.1% of scaffolds, or one error per 3.2 Mb, highlighting the importance of ultra-high density linkage mapping.

## Concluding remarks

The combination of whole genome shotgun sequencing and linkage mapping by skim sequencing produced a better genome assembly than both the chromosome arm-based assembly and a previously described whole genome shotgun sequencing assembly approach with 5× 454 sequences (limited by low sequencing depth) [9]. Increasing sequencing depth from the current 30× to 90× that is normally practiced for whole genome sequencing using short reads would further improve the assembly. Even with the current fragmented assembly, it serves as a working reference for mining target genes and for genome-wide association studies through re-sequencing of a collection of diverse germplasm.

This method is not only applicable and beneficial for other large allopolyploid genomes like rye and oats. It should also be applied to draft genome sequencing of all species, both diploid and polyploid. Genotyping by skim sequencing of individual genomes is affordable and the sequencing depth could be increased from 0.2× in rice and 1.4× in wheat to 2× to 4× in any genome [4,10]. The increased depth would not enhance the map resolution, but it would increase power and accuracy for the correction of misassembled scaffolds.

## References

[1] Jiao Y, Wickett NJ, Ayyampalayam S, Chanderbali AS, Landherr L, Ralph PE (2011). Ancestral polyploidy in seed plants and angiosperms. Nature.

[2] Wood TE, Takebayashi N, Barker MS, Mayrose I, Greenspoon PB, Rieseberg LH (2009). The frequency of polyploid speciation in vascular plants. Proc Natl Acad Sci U S A.

[3] Ramsey J, Schemske DW (1998). Pathways, mechanisms, and rates of polyploid formation in flowering plants. Annu Rev Ecol Syst.

[4] Chapman JA, Mascher M, Bulu A, Barry K, Georganas E, Session A, et al. In favor of a whole-genome shotgun: assembling and anchoring the hexaploid bread wheat genome. Genome Biol. 2014;16:582 10.1186/s13059-015-0582-8 PMC4373400 25637298

[5] Choulet F, Alberti A, Theil S, Glover N, Barbe V, Daron J (2014). Structural and functional partitioning of bread wheat chromosome 3B. Science.

[6] International Wheat Genome Sequencing Consortium (2014). A chromosome-based draft sequence of the hexaploid bread wheat (*Triticum aestivum*) genome. Science.

[7] Chapman JA, Ho I, Sunkara S, Luo S, Schroth GP, Rokhsar DS (2011). Meraculous: de novo genome assembly with short paired-end reads. PLoS One.

[8] Chalhoub B, Denoeud F, Liu S, Parkin IAP, Tang H, Wang X (2014). Early allopolyploid evolution in the post-Neolithic *Brassica napus* oilseed genome. Science.

[9] Brenchley R, Spannagl M, Pfeifer M, Barker GL, D’Amore R, Allen AM (2012). Analysis of the bread wheat genome using whole-genome shotgun sequencing. Nature.

[10] Huang X, Feng Q, Qian Q, Zhao Q, Wang L, Wang A (2009). High-throughput genotyping by whole-genome resequencing. Genome Res.

